# A Preliminary Investigation into the Accuracy of 3D Modeling and 3D Printing in Forensic Anthropology Evidence Reconstruction†,‡

**DOI:** 10.1111/1556-4029.13917

**Published:** 2018-10-08

**Authors:** Rachael M. Carew, Ruth M. Morgan, Carolyn Rando

**Affiliations:** ^1^ Department of Security and Crime Science University College London 35 Tavistock Square London U.K WC1H 9EZ; ^2^ Centre for the Forensic Sciences University College London 35 Tavistock Square London U.K WC1H 9EZ; ^3^ Institute of Archaeology University College London 31‐34 Gordon Square London U.K WC1H 0PY

**Keywords:** forensic science, forensic anthropology, 3D printing, additive manufacturing, computed tomography, evidence reconstruction, metrology

## Abstract

There is currently no published empirical evidence‐base demonstrating 3D printing to be an accurate and reliable tool in forensic anthropology, despite 3D printed replicas being exhibited as demonstrative evidence in court. In this study, human bones (n = 3) scanned using computed tomography were reconstructed as virtual 3D models (n = 6), and 3D printed using six commercially available printers, with osteometric data recorded at each stage. Virtual models and 3D prints were on average accurate to the source bones, with mean differences from −0.4 to 1.2 mm (−0.4% to 12.0%). Interobserver differences ranged from −5.1 to 0.7 mm (−5.3% to 0.7%). Reconstruction and modeling parameters influenced accuracy, and prints produced using selective laser sintering (SLS) were most consistently accurate. This preliminary investigation into virtual modeling and 3D printer capability provides a novel insight into the accuracy of 3D printing osteological samples and begins to establish an evidence‐base for validating 3D printed bones as demonstrative evidence.

Additive manufacturing can be utilized by forensic anthropologists, pathologists, and radiographers to create physical models of skeletal injuries, which in turn can aid in the explanation of trauma, in terms of its etiology, as well as the mechanism and mode of injury 1, 2, 3. Three‐dimensional (3D) visual aids are believed to be easier to understand in comparison with photographs for explaining complex or disturbing medical information to jurors and medical laypeople 4, and as such, 3D printed replicas have been used as supporting evidence in courts of law in several countries 5, 6, 7. However, there is currently an absence of a clear and demonstrable evidence‐base to show 3D printed replicas of osteological evidence to be accurate representations.

As a first step toward validating 3D printed replicas for use as evidence, this experimental study compares known bone samples with virtual and physical 3D models of those bones to investigate the accuracy and capabilities of these reconstruction techniques. This study addresses the impact of different printer types and assesses the level of accuracy that is attainable in producing replicas of bones. Thus, the findings may contribute to the creation of an empirical evidence‐base that can establish 3D printing as a valuable tool in the forensic reconstruction process 8, 9.

## Demonstrative Evidence in Court

Digital methods of presenting evidence are particularly useful in forensic anthropology, as human remains cannot be taken into court since they could be disturbing or hazardous, and could potentially prejudice a jury 4. Digital methods have become increasingly popular and have been used in place of traditional photographs for demonstrating evidence in court for a number of years 4. It can be argued that both photographs and 3D virtual models may not always provide accurate representations of their original subject. First, subjects can be distorted via the light or angle used in a photograph or a virtual rendering; second, when presenting a 3D object such as a bone as a 2D image, whether as a photograph or a virtual model, depth and spatial information is immediately lost 2, 10; and third, virtual 3D models are stereoscopic, meaning that they only give the illusion of depth. A novel way to address these problems has been the introduction of 3D printed replicas: a physical 3D object that has depth, haptic, and spatial characteristics 2.

3D printed evidence is a natural development of virtual anthropology 2, 11, 12, and recent reports have highlighted the value of forensic imaging and 3D printing as demonstrative aids in court, while also highlighting the need for empirically sourced data to support such tools 13, 14. Indeed, there is a drive toward creating evidence‐based approaches across the forensic sciences. These approaches draw on the existing knowledge bases of parent disciplines and require research that can provide specific forensic “evidence” bases that can then be used to underpin each stage of the forensic process, from crime scene to court 9, 15, 16, 17, 18, 19, 20. This enables the reconstruction process to be presented transparently and provides a means of demonstrating how a specific conclusion has been reached.

A number of cases have been identified in the published literature and in the media where 3D printed representations of bone have been presented as demonstrative evidence. Two cases in the United Kingdom 5, 6 and one in Germany 7 utilized 3D prints in court, and two others in the United Kingdom and Poland, where a print was utilized by the prosecutor but not presented in court 3, 21. The issue of introducing new technology in court was highlighted in a case tried in the United Kingdom in 2016 6, whereby the defense counsel cast doubt on the reliability of a 3D printed cranium. At trial, it was stated that the manufacturing process had not been validated in a forensic context and was simply an interpretation, thereby undermining the weight of the evidence 6. 3D printed exhibits are already being used in U.K. courts as demonstrative evidence since the laws in England and Wales permit the use of novel technologies in court on the basis of expert opinion. It is important to differentiate between demonstrative evidence and demonstrative aids, and the rules of evidence governing their use in court. Demonstrative (or substantive) evidence is evidence that is admitted into court and as such is subject to the relevant rules of evidence/admissibility, for example The Criminal Procedure Rules (CrimPR part 19, Expert Evidence) 22 in the United Kingdom, and the Daubert/Frye criteria of admissibility 4, 23 and the Federal Rules of Evidence (611 and 1006, regarding admission of demonstrative exhibits) in the USA 24, 25. A key factor of demonstrative evidence is that it can be reviewed by the jury during deliberations 24, 25. In contrast, demonstrative (or illustrative) aids are visual aids used in court to help explain admitted evidence (e.g., expert testimony) and to assist jurors in understanding factual issues; demonstrative aids are not themselves admitted into evidence and have no probative value 24, 25. Additionally, demonstrative aids are not governed by such stringent rules of admissibility. While it would be extremely valuable to have an evidence‐base in place that underpins the validation of 3D prints as demonstrative aids—if 3D prints were to be used as evidence in court—it would be vital to be able to establish the accuracy, reliability and preservation of the prints to meet any rules of admissibility. The research here addresses the lack of validation present for 3D printing bones that is applicable when used as either demonstrative evidence or a demonstrative aid.

## Digitising and Reproducing Bone Specimens

While there are numerous techniques available for digitising bone specimens, computed tomography (CT) scanning offers several advantages over digital surface scanning techniques. CT is noncontact and noninvasive, which negates any need to un‐package, clean or macerate human remains (which could destroy potential forensic evidence). CT can be used with both living and deceased individuals, and since it records volumetric data, CT can be employed in the examination of antemortem skeletal injuries and not simply surface injuries as with other scanning technologies. It was for these reasons that CT was selected as the digitising method in this study.

CT is widely accepted to be a robust technique that can accurately record the dimensions of its subject, including in anthropology 1, 26, 27, 28, 29, 30. Nevertheless, metric differences can stem from multiple sources of error including the following: scanning parameters, scanner reconstruction algorithms, surface reconstruction parameters, as well as, printing resolution, landmark selection and ruler positioning 31, 32. Stull (2014) state that an error range of ±2.0 mm is acceptable for anthropological assessments 33, while Langley (2018) state that acceptable technical error of measurement (TEM) values are <1.5% for intraobserver error and <2.0% for interobserver error 34. A review of 3D print accuracy in the literature (comparing 3D prints against the dry skull or 3D CT model) observed that the percentage mean differences reported ranged from 0.56% to 4.7% 35. Additionally, 3D printing resolution is reported to be as low as 0.05 to 0.1 mm 2, 36, which is greater than the resolution generally used in clinical CT scans 2, 36. Furthermore, these differences have been identified as “clinically negligible” 12.

Rapid prototyping includes both additive and subtractive manufacturing, with the majority of current popular techniques using additive manufacturing and often being referred to as “3D printing” 36; this term is advocated for continued consistency 37. 3D printing techniques are classified into vat polymerization, material extrusion, material jetting, binder jetting, powder bed fusion, sheet lamination and directed energy deposition by The American Society for Testing and Materials 12, 36. Each of these techniques may involve different materials and various printer manufacturers, resulting in many 3D printers to choose from. There are advantages and limitations to each technique 12. The primary considerations for this current study were the printing resolution, type of material, build size limit, and the use and removal of support structures.

## Emerging Challenges

3D printing in forensic anthropology is an emerging multidisciplinary topic, with major challenges that need to be addressed through empirical research. Priority issues include validation of the 3D modeling and printing processes, establishing which 3D printing methods produce accurate and realistic replicas, and the exploration and quantification of the evidential impact when using 3D techniques for demonstration of evidence. Only by addressing these novel issues can an evidence‐base be generated to facilitate the use of 3D printed replicas as evidence in court, whereby 3D prints can be used reliably and transparently. The aim of this preliminary investigation was to examine the level of accuracy demonstrated when producing a 3D printed replica of bone, and to evaluate the reliability of replicas from different 3D printers.

## Materials and Methods

### Data Acquisition

**Figure 1:**
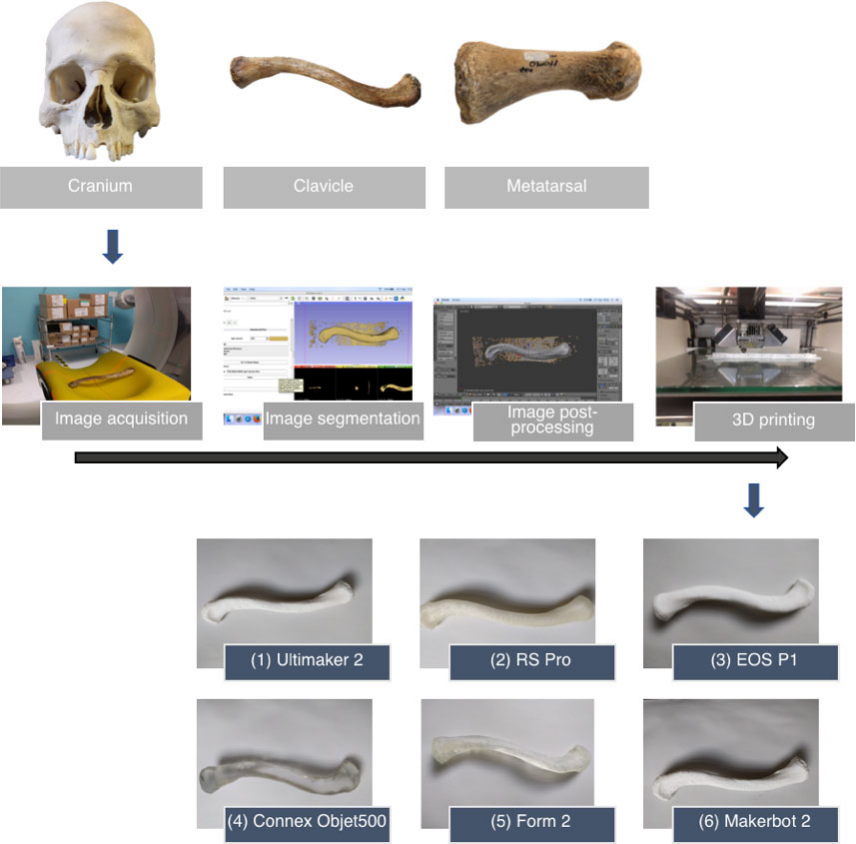
Overview of the 3D printing process. Upper: samples; centre: image acquisition (CT scanning), image segmentation (using 3D Slicer), image postprocessing (using Blender), and 3D printing (using Ultimaker 2); lower: 3D prints of clavicle using six different printers (see Table 1 for printer details). [Color figure can be viewed at wileyonlinelibrary.com]

In this study, archeological human bone specimens (n = 3) that were dry and in good condition were loaned from the University College London (UCL) Institute of Archaeology. First, a cranium was chosen due to its complex, large structure, with many measurement points available; second, a clavicle and first metatarsal were selected, as these are similar in structure to long bones but are smaller and thus more affordable to print multiple times. An overview of the production process is presented in Fig. 1. The bones were scanned at University College London Hospital (UCLH) by an on‐site clinical radiographer, using a Toshiba Aquilion ONE Vision Edition (Canon Medical Systems Corporation, Otawara, Japan) helical multidetector CT scanner. Scanning parameters were 0.5 mm slice thickness at 0.25 mm intervals, 120 kVp, data collection diameter 240 mm, mAs 266, 204 and 234, and field of view (FOV) 220.321, 162.187 and 79.687 mm (both, respectively, per bone), with bone and soft tissue reconstructions (Bone Sharp FC30 and Soft Tissue Standard FC08). The CT images were saved as Digital Imaging and Communications in Medicine (DICOM) data and transferred to a compact disk (CD).

### Reconstruction

The DICOM data were reconstructed using 3D Slicer (3D Slicer, Brigham Women's Hospital, Boston, MA, US) 38, a free, open‐source, cross‐platform program. The soft tissue CT reconstruction was segmented using threshold values (based on the radio‐density of the structure), and the level adjusted to include the desired proportion of bone (so as to not lose detail) 12, 39, 40. A surface model was generated that was automatically smoothed (using 3D Slicer's Editor Module and Make Model tool), and then exported as an STL (stereolithography, or standard tessellation language) file. The STL file (“3D virtual model”) was subsequently opened in Blender (Stichting Blender Foundation, Amsterdam, the Netherlands), any background artefacts were deleted, and the model “smoothed” by a factor of 0.5 iterated 10 times and then 20 times, producing three models from the same bone: the original (Virtual Model A); one smoothed × 10 (Virtual Model B); and one smoothed × 20 (Virtual Model C). For comparison, a model was also generated without 3D Slicer's auto‐smoothing (“nonauto‐smoothed”, Virtual Model D).

**Figure 2:**
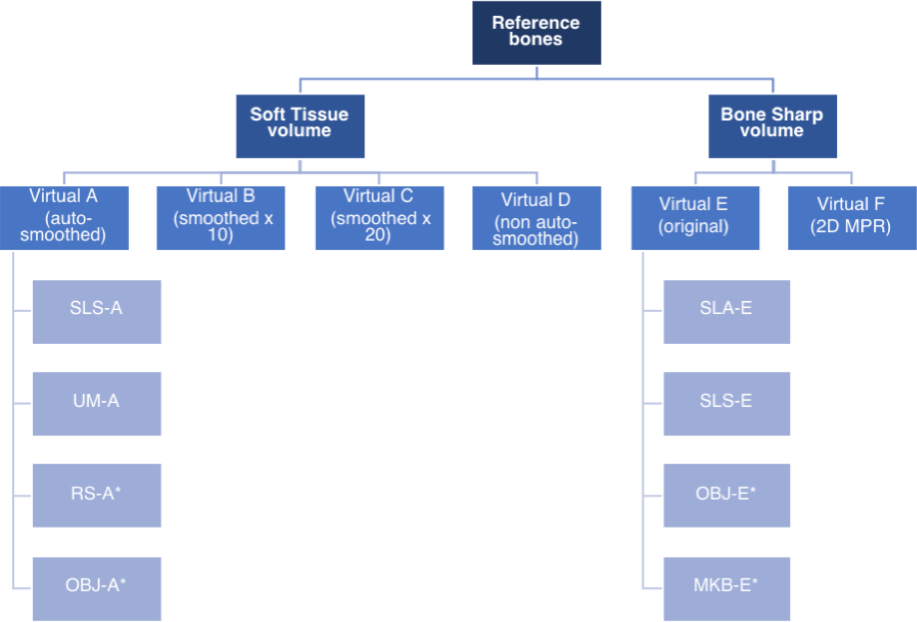
Model production flow chart, showing virtual models and 3D replicas. See Table 1 for printer details. *Clavicle and metatarsal only. [Color figure can be viewed at wileyonlinelibrary.com]

Virtual Model E was later generated using the Bone Sharp volumetric data to obtain an additional model similar to Virtual Model A (auto‐smoothed without additional smoothing). A model production flow chart is illustrated in Fig. 2. The STL files were prepared for printing and printed on six different printers incorporating material extrusion (fused deposition modeling, FDM), powder bed fusion (selective laser sintering, SLS), material jetting, and vat polymerization (stereolithography, SLA) techniques; printing parameters are detailed in Table 1.

**Table 1:** 3D printing parameters and materials

Printer		Printing process	Acronym	Material	Layer height (mm)	Cranium printed	Support structure removal
1	Ultimaker 2 (Ultimaker B.V. Geldermalsen, the Netherlands)	Fused Deposition Modeling (FDM)	UM	White PLA^a^	0.100 (cranium), 0.06 (clavicle), 0.200 (metatarsal)	Yes	Yes, manual with pliers
2	RS Pro (RS Components Ltd, UK)	Fused Deposition Modeling (FDM)	RS	Clear PLA^a^	0.200	No, build volume too small	Yes, manual with pliers
3	EOS P1 (EOS GmbH Electro Optical Systems, Germany)	Selective Laser Sintering (SLS)	SLS	White nylon powder (PA2200)	0.100	Yes	No
4	Connex 3 Objet500 (Stratasys Ltd, Eden Prairie, MN, US)	Material jetting with UV curing	OBJ	Vero Clear resin	0.032	No, too costly	No
5	Form 2 (Formlabs Inc, Somerville, MA, US)	Stereolithography (SLA) with laser curing	SLA	Clear resin	0.005	Yes, but split into four parts	Yes, manual with pliers
6	MakerBot 2 (MakerBot Industries, New York, NY, US)	Fused Deposition Modeling (FDM)	MKB	White PLA^a^	0.200	No, build volume too small	Yes, manual with pliers

a Polylactic acid (polyester plastic)

### Osteometric Measurements

Linear osteometric measurements (detailed in Table 2) of the cranium (*n *=* *26), clavicle (*n *=* *3) and metatarsal (*n *=* *3) were obtained from the reference bones, the 3D virtual models, and the 3D printed replicas 41, 42. Measurements on the reference bones and printed material were taken using manual sliding calipers (to the nearest millimeter) and digital sliding calipers (to the nearest hundredth millimeter). One observer (with approximately 5 years of experience in taking measurements of this type) collected the initial measurement data (gold standard) from each source to minimize potential bias.

**Table 2:** Osteometric measurements and locations 41, 42. PA (posterior‐anterior), SI (superior‐inferior), LM (lateral‐medial)

	Acronym	Measurement	Point A	Symbol	Point B	Symbol
Cranium	GOL^a^	Maximum cranial length	Glabella	g	Opisthocranion	op
	XCB^a^	Maximum cranial breadth	Euryon	eu	Euryon	eu
	ZYB^a^	Bizygomatic breadth	Zygion	zy	Zygion	zy
	BBH^a^	Maximum cranial height	Basion	ba	Bregma	b
	BNL^a^	Cranial base length	Basion	ba	Nasion	n
	BPL	Basion‐prosthion length	Basion	ba	Prosthion	pr
	MAB	Maxillo‐alveolar breadth	Ectomalare	ecm	Ectomalare	ecm
	MAL	Maxillo‐alveolar length	Prosthion	pr	Alveolon	al
	AUB	Biauricular breadth	Root of zygomatic process	–	Root of zygomatic process	–
	UFHT	Upper facial height	Nasion	n	Prosthion	pr
	WFB	Minimum frontal breadth	Frontotemporale	ft	Frontotemporale	ft
	UFBR	Upper facial breadth	Fronto‐zygomatic suture	–	Fronto–zygomatic suture	–
	NLH	Nasal height	Nasion	n	Nasospinale	ns
	NLB	Nasal breadth	Alare	al	Alare	al
	OBB	Orbital breadth	Dacryon	d	Ectoconchion	ec
	OBH	Orbital height	Superior margin	–	Inferior margin	–
	EKB	Biorbital breadth	Ectoconchion	ec	Ectoconchion	ec
	DKB	Interorbital breadth	Dacryon	d	Dacryon	d
	FRC	Frontal chord	Nasion	n	Bregma	b
	PAC	Parietal cord	Bregma	ba	Lambda	l
	OCC	Occipital chord	Lambda	l	Opisthion	o
	FOL	Foramen magnum length	Opisthion	o	basion	ba
	FOB	Foramen magnum breadth	Most lateral point of foramen magnum	–	Most lateral point of foramen magnum	–
	MDH	Mastoid length (left)	Porion	po	Mastoidale	ma
	ASB	biasterion breadth	Asterion	–	Asterion	–
	ZMB	Zygomaxillary breadth	Zygomaticomaxillary suture	zs	Zygomaticomaxillary suture	zs
Clavicle	CML	Clavicle maximum length	Most distal point of clavicle	–	Most proximal point of clavicle	–
	DMS PA	Mid‐shaft diameter	Posterior surface	–	Anterior surface	–
	DMS SI	Mid‐shaft diameter	Superior surface	–	Inferior surface	–
Metatarsal	MML	Metatarsal maximum length	Most distal point of metatarsal	–	Most proximal point of metatarsal	–
	MTD LM	Mid‐shaft diameter	Lateral surface	–	Medial surface	–
	MTD SI	Mid‐shaft diameter	Superior surface	–	Inferior surface	–

a Taken using spreading calipers, remainder using sliding calipers.

3D Slicer's Fiducial and Ruler tools were utilized for the virtual data collection, with the software allowing precision to the nearest tenth of a millimeter. The virtual 3D model was manually rotated on‐screen to view landmarks and obtain traditional anthropological linear measurements from the standard views (*norma frontalis, norma occipitalis, norma lateralis* (left and right), and *norma basilaris*) 43. Data were also obtained from a two‐dimensional (2D) multiplanar reconstruction (MPR) for comparison (Virtual Model F). 3D Slicer's Fiducial and Ruler tools were used to obtain linear data from the sagittal, coronal, and axial views 30. Data collection was repeated for reference bones (*n *=* *9), virtual models (*n *=* *9, except models Virtual E and F, where *n *=* *3), and 3D prints (*n *=* *3); each set was obtained blinded to previous results and taken on separate days.

Five additional observers, all archeology/forensic anthropology doctoral students, measured the reference bones, two of the virtual models (Virtual Model A and Virtual Model E) and four of the 3D printed replicas (SLS‐A, UM‐A, SLA‐E, and SLS‐E), using the same methods as above for data collection. The observers each have experience taking osteometric measurements through study/research and two through employment, additionally, one observer had previous experience with virtual models/CT data/3D Slicer, while none had experience of 3D printed replicas. The observers did not take repeat measurements, and they were instructed to measure the reference bones last. Instructions informed the observers which measurement points and instruments to use, and an online video detailed how to take measurements using 3D Slicer (using a different skeletal element).

### Analyses

Intraobserver reliability, interobserver reliability, and accuracy analyses were performed using Microsoft Excel version 16.9 for Mac (Microsoft, Redmond, WA, US). TEM was not suitable for this study due to the limited sample size; therefore, the accepted error range of ±2.0 mm, provided by Stull (2014), was employed to evaluate the differences. To evaluate intraobserver reliability, the initial dataset (reference bones, virtual models and 3D printed replicas) was assessed for repeatability using descriptive statistics (standard deviation and variance) and within‐subject standard deviation (wSD; square root of the average variances) 30. Using the mean values for each dataset, the metric difference (mean observed value minus mean reference value) and percentage difference (metric difference divided by mean reference value, all multiplied by 100) were calculated to assess the accuracy of the data between methods and identify any trends for each bone, for the virtual and 3D print models 31, 33, 44. Since the impact of an error of 2 mm is different at different orders of measurement 33, percentage differences were included as these are independent of the size of the measurement. As an indicator for interobserver reliability, metric differences and percentage differences were calculated subtracting the observers’ value from the initial mean data (the gold standard).

## Results

**Figure 3:**
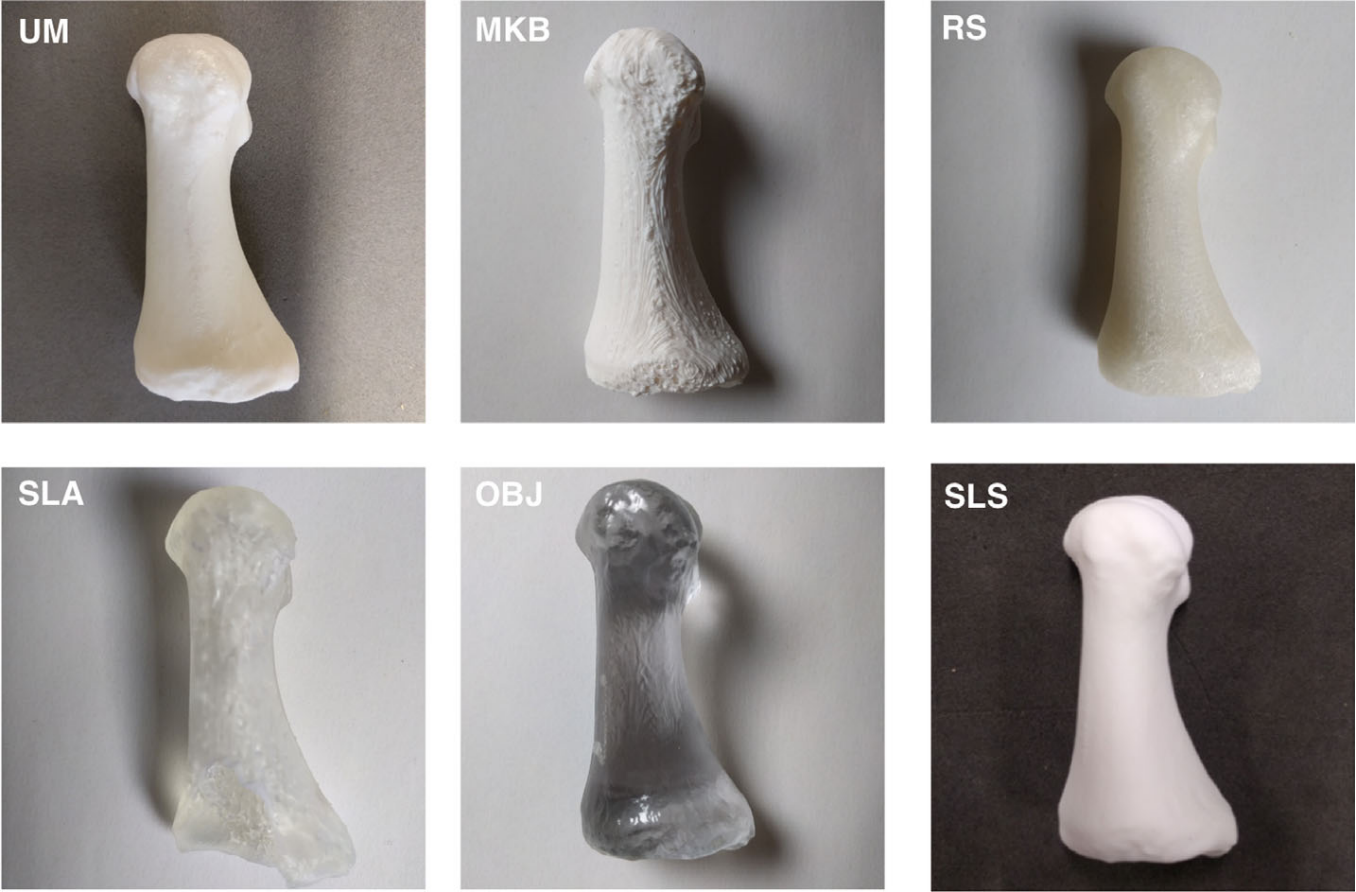
Lateral views of six 3D printed replica metatarsals. [Color figure can be viewed at wileyonlinelibrary.com]

**Figure 4:**
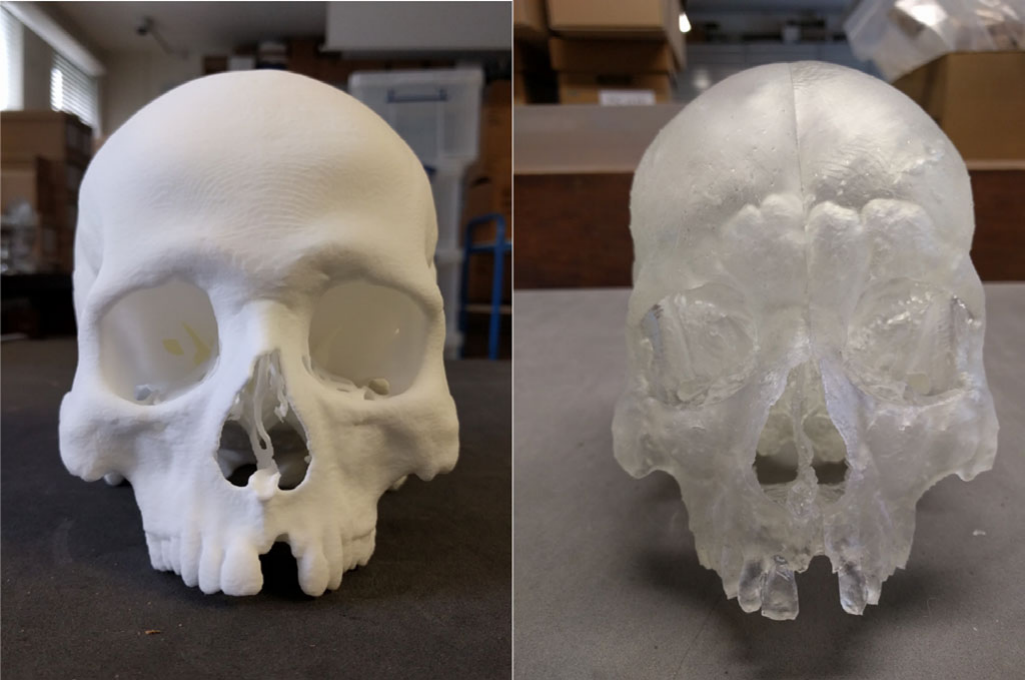
3D printed cranium replicas, printed using SLS (left), SLA (right). [Color figure can be viewed at wileyonlinelibrary.com]

**Figure 5:**
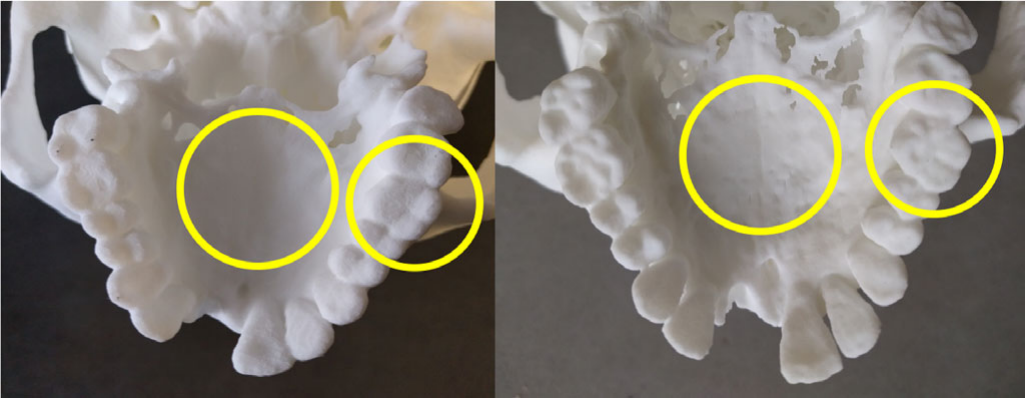
SLS printed cranium replica from Virtual Model A (left), and from Virtual Model E (right). Illustrating the level of detail of palatine suture (large circle) and dentition (small circle). [Color figure can be viewed at wileyonlinelibrary.com]

**Figure 6:**
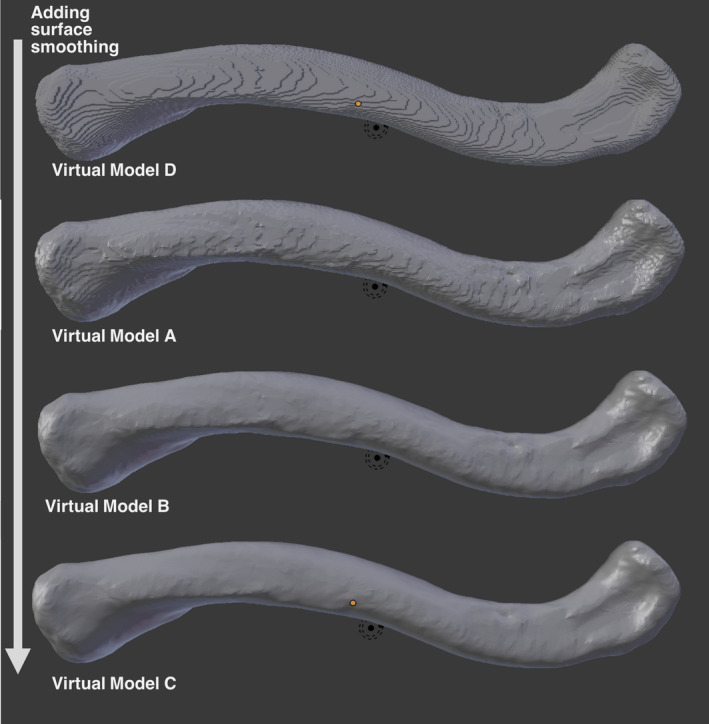
Virtual models viewed in Blender with different levels of surface smoothing. [Color figure can be viewed at wileyonlinelibrary.com]

**Figure 7:**
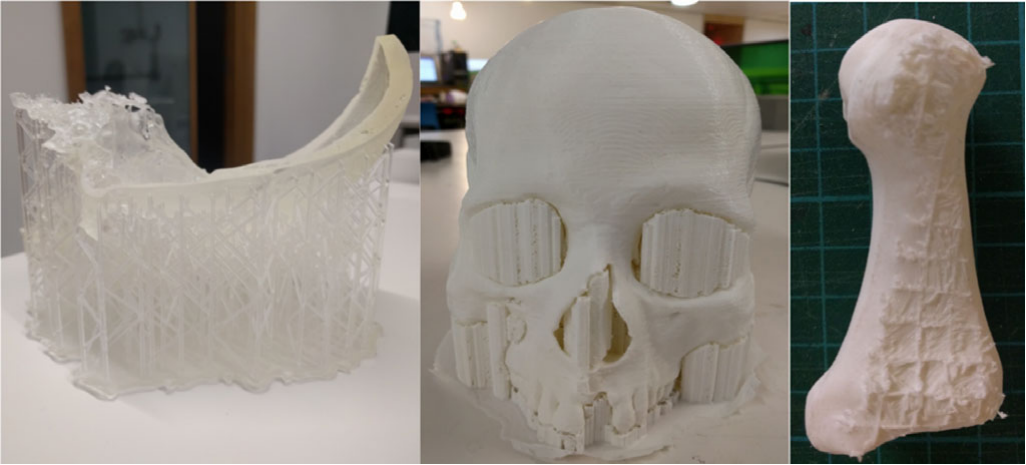
Support structures from 3D printing shown on SLA cranium section (left), UM cranium (centre), and residual supports after removal on UM metatarsal (right). [Color figure can be viewed at wileyonlinelibrary.com]

Five 3D virtual models were generated (A‐E) and eight 3D prints replicated, four each from Virtual Model A and Virtual Model E (Fig. 2), using six different 3D printers. The variation in surface quality across printers was markedly different (Figs 3 and 4), as was the variation between CT reconstruction volumes (Fig. 5) and the alteration to appearances with surface smoothing (Fig. 6). The 3D printing support structures varied with printer type and left residual attachments on the prints (Fig. 7).

### Repeatability

Low average standard deviations (SD) and variances (Var) were found for reference data, particularly the two smaller bones (clavicle and metatarsal). The within‐subject standard deviation (wSD) for the reference bones data ranged from 0.3 mm (0.9 mm 95% repeatability) to 0.9 mm (2.5 mm 95% repeatability). The virtual model intraobserver data gave wSD values that ranged from 0.1 mm (0.3 mm 95% repeatability) to 2.2 mm (6.1 mm 95% repeatability), and for 3D print data from 0.1 mm (0.2 mm 95% repeatability) to 1.7 mm (4.8 mm 95% repeatability) (Tables 3 and 4).

**Table 3:** Intraobserver reference (bolded) and virtual model within‐subject standard deviations (wSD) and 95% repeatability (% R) in mm

Element	Reference		Virtual A		Virtual B		Virtual C		Virtual D		Virtual E		Virtual F	
	wSD	% R	wSD	% R	wSD	% R	wSD	% R	wSD	% R	wSD	% R	wSD	% R
Cranium	**0.9**	**2.5**	1.4	4.0	1.5	4.1	2.2	6.1	1.6	4.5	0.4	1.1	0.7	1.9
Clavicle	**0.3**	**0.9**	0.8	2.3	0.9	2.5	1.6	4.5	0.7	2.0	0.2	0.4	0.4	1.0
Metatarsal	**0.3**	**0.9**	0.6	1.7	0.5	1.3	0.8	2.3	0.9	2.6	0.1	0.3	0.2	0.6

**Table 4:** Intraobserver 3D print within‐subject standard deviations (wSD) and 95% repeatability (% R) in mm

Element	Print SLS‐A		Print UM‐A		Print RS‐A		Print OBJ‐A		Print SLS‐E		Print SLA‐E		Print OBJ‐E		Print MKB‐E	
	wSD	% R	wSD	% R	wSD	% R	wSD	% R	wSD	% R	wSD	% R	wSD	% R	wSD	% R
Cranium	1.2	3.3	1.0	2.9	–	–	–		0.8	2.3	1.7	4.8	–	–	–	–
Clavicle	0.3	0.9	0.2	0.5	0.2	0.7	0.1	0.2	0.1	0.4	0.2	0.5	0.1	0.4	0.2	0.6
Metatarsal	0.2	0.6	0.1	0.3	0.1	0.4	0.1	0.4	0.1	0.2	0.1	0.2	0.2	0.5	0.1	0.4

### Accuracy

Mean differences in the initial mean dataset (Table 5) ranged from −0.4 to 1.2 mm for the virtual model data, with percentage differences of −0.4% to 12.0%, and from −0.2 to 1.2 mm for 3D print data, with percentage differences from −0.2% to 9.9%. For the cranium data, mean differences in virtual models and 3D prints were all within 1.0 mm, with percentage differences of ±2.0%. For the smaller bones, mean differences were both within ±1.2 mm, and the percentage differences within 12.0% for the clavicle and within ±5.0% for the metatarsal.

**Table 5:** Initial dataset: mean (mm), maximum (mm) and percentage (%) differences from reference bones

	Cranium			Clavicle			Metatarsal		
	Mean (mm)	Maximum (mm)	Percentage (%)	Mean (mm)	Maximum (mm)	Percentage (%)	Mean (mm)	Maximum (mm)	Percentage (%)
Virtual‐A	0.8	2.9	1.3	1.1	2.3	12.0	1.1	2.1	3.7
Virtual‐B	0.7	2.6	1.3	0.6	1.7	8.5	0.8	1.5	2.5
Virtual‐C	0.9	5.1	1.7	0.3	2.8	9.1	0.8	1.8	2.0
Virtual‐D	0.8	3.8	1.5	1.0	2.0	10.6	1.2	2.3	3.7
Virtual‐E	0.3	2.8	0.9	−0.1	0.4	1.3	0.2	0.8	−0.4
Virtual‐F	0.2	9.6	0.3	−0.4	1.0	0.7	0.8	1.4	3.0
Print SLS‐A	0.6	2.7	0.6	1.1	1.9	9.9	0.9	1.2	3.7
Print UM‐A	0.7	4.0	0.8	1.0	1.8	9.8	1.0	1.3	4.9
Print SLA‐E	0.3	3.1	0.2	0.3	0.5	1.7	0.4	1.3	0.6
Print SLS‐E	0.0	3.0	−0.2	0.0	0.1	0.3	0.3	1.4	‐0.1
Print OBJ‐A	–	–	–	1.2	1.7	9.2	1.1	1.5	4.5
Print RS‐A	–	–	–	1.2	1.5	9.7	0.9	1.4	4.3
Print OBJ‐E	–	–	–	0.3	0.6	2.1	0.3	1.3	0.0
Print MKB‐E	–	–	–	0.0	0.7	2.4	0.2	0.6	0.9

### Interobserver

Two cranial measurements were excluded from the interobserver data due to errors made during data collection procedures (GOL and ASB). The interobserver virtual data differences ranged from −3.6 to 0.0 mm, with percentages differences of −5.3 to 0.6% (Table 6). The 3D print differences ranged from −5.1 to 0.7 mm, with percentage difference of −5.2 to 0.7% (Table 7).

**Table 6:** Virtual model interobserver data: average differences (mm) and average percentage differences (%) for all bones

Observer	Reference		Virtual‐A		Virtual‐E	
	Difference (mm)	Percentage Difference (%)	Difference (mm)	Percentage Difference (%)	Difference (mm)	Percentage Difference (%)
1	−1.0	−1.0	−2.1	−3.2	−1.9	−2.4
2	−3.6	−4.0	−1.2	−0.5	0.0	0.6
3	−1.5	−1.1	−2.6	−2.7	−2.4	−2.2
4	−4.6	−4.0	−1.5	−0.9	−0.4	2.5
5	−1.3	−0.5	−3.6	−5.3	−3.1	−3.8

**Table 7:** 3D print interobserver data: average differences (mm) and average percentage differences (%) for all bones

Observer	Print SLS‐A		Print UM‐A		Print SLS‐E		Print SLA‐E	
	Difference (mm)	Percentage Difference (%)	Difference (mm)	Percentage Difference (%)	Difference (mm)	Percentage Difference (%)	Difference (mm)	Percentage Difference (%)
1	−0.7	0.4	−0.4	0.7	−0.7	0.4	−1.2	−0.6
2	−3.6	−3.1	−4.0	−4.0	−3.9	−3.8	−3.8	−3.9
3	−1.8	−1.3	−1.6	−0.5	−1.4	−0.6	−1.6	−1.4
4	−4.8	−4.2	−5.2	−5.1	−3.2	−2.3	−4.5	−4.3
5	−1.6	−0.2	−1.8	−0.2	−2.1	−0.9	−1.7	−0.9

## Discussion

Exploration of the metrology of 3D printing and an understanding of the factors influencing model production is paramount to validating 3D printing in forensic anthropology. The results from this study found good intraobserver reliability and indicate good accuracy. The data resulted in mean differences ranging from −0.4 to 1.2 mm (−0.4% to 12.0%) for the virtual model data, and from −0.2 to 1.2 mm (−0.2% to 9.9%) for 3D print data. The error recorded is comparable to that previously reported for virtual 3D model accuracy 33, 43, 45, 46, as well as 3D print accuracy 35, 44, 47.

### Repeatability

Intraobserver measurement error (wSD) was within 1.0 mm for the reference data and less than 2.0 mm for all datasets except Virtual Model C for cranium, this being in line with those previously reported 30, 43 indicating good repeatability overall. Variation in reliability was reported between the virtual models, Virtual Model A data showed low reliability (wSD > 2.0 mm), and data from Virtual Models A, B and C exhibited high 95% repeatability (>4.0 mm) for cranium data. The 3D print data showed good reliability with wSD values within 2.0 mm (highest wSD 1.7 mm for SLA‐E cranium data); thus, the 3D print data were of comparable reliability to the reference bones data.

### Accuracy

The 3D virtual models and 3D prints produced were on average accurate to the source bones, with mean differences of ±1.2 mm and therefore, within the accepted level of ±2.0 mm 33. Virtual Model E (Bone Sharp volume) was consistently more accurate than Virtual Model A (Soft Tissue volume), for both the virtual model and subsequent 3D print data. Additionally, the superior surface detail produced from Virtual E (Fig. 5) corroborates previous findings that found volume reconstruction algorithms to affect model accuracy 45; consideration should be given when choosing reconstruction filters. Virtual Model F (2D MPR) had particularly high maximum differences (9.6 mm) for cranium data, indicating that obtaining measurements from a 2D method is less reliable than measuring from a 3D model for large complex structures. This concurs with previous work that found 3D models to be superior for visualizing morphological features 48 and citing the 2D image distortion issues previously mentioned.

There was no observable difference in accuracy between Virtual Model A (original model) and Virtual Model D (nonauto‐smoothed model). It is thought that this automatic smoothing in 3D Slicer is perhaps useful for appearance, to smooth the CT slices, but it is not significant enough to affect the model metrically (at the level considered in this study). Similarly, the additional smoothing applied in Blender does not have an observable effect on accuracy for Virtual Model B (smoothed × 10) versus Virtual Model C (smoothed ×20). However, a variation in surface quality is visible with increasing smoothing (Fig. 6). Initial smoothing appears to aid surface quality by removing stepping from the CT scan slices; however, surface morphology appears to be at risk of being altered when further additional smoothing (×20) is applied (Virtual Model C). Further research is needed to investigate smoothing algorithms and care must be taken to avoid distorting the original dimensions 32.

3D printer layer heights ranged from 0.005 to 0.5 mm, and CT pixel sizes were calculated to 0.4 mm for cranium, 0.3 mm for clavicle, and 0.2 mm for metatarsal. Consequently, printer resolution was always greater than the CT resolution for cranium and clavicle, and greater, or the same, resolution for the metatarsal. As a result, the resolution of the 3D printers should not have affected the accuracy of the 3D prints, and the major influencing factor was the reconstruction algorithms and segmentation protocol, agreeing with previous conclusions 12, 47.

Cranium datasets generally exhibited lower reliability and accuracy compared with the smaller bones, this likely due to the cranium's more complex structure and large curved surface, which makes it both more difficult to fully visualize on a computer screen, and more challenging for a 3D printer to successfully print. While percentage differences were higher for the clavicle and metatarsal, this can be attributed to the small measurement size, c.10.0 mm diameter compared to the c.138.0 mm length. 3D Slicer proved sufficient for producing accurate virtual models; however, with numerous software packages available for modeling CT data, it is important to consider the impact of the software used and the algorithms inherent within them. Hodgdon et al. 12 suggest using software approved by the US Food and Drug Administration (FDA) to ensure model accuracy. The FDA has issued guidance on 3D printing in medical contexts to ensure safe and effective use 12, and these could prove useful for ensuring the accuracy when producing 3D prints for use in a court of law.

### Interobserver Reliability

There is no apparent trend in measurement error between the type of model or print, but differences were identified between the observers. Observer 1 had average differences across all bones of ±1.0 mm, Observer 2 ± 3.0 mm, Observer 3 ± 1.5 mm, Observer 4 ± 5.0 mm, and Observer 5 ± 2.0 mm for 3D print data. This could be explained by the level of experience of the observers, suggesting that more training is needed, particularly for virtual data collection. Indeed, only one of the additional observers had prior experience in this area. Average differences across all bones for 3D print data were within the accepted limit of ±2.0 mm 33 and <2.0% 34 for three of the five additional observers. Interobserver variability rates were higher than intraobserver variability, concordant with the scientific literature 31, 33, 34, and interobserver error was higher for the measurements obtained using manual spreading calipers, possibly due to the lower precision of the instrument, or greater difficulty using these calipers.

### 3D Printing Techniques

Replica build times were not recorded in this study but ranged from several hours to four days (for a full adult cranium using FDM). Production cost also varies significantly between methods, with print costs ranging from c.£20 to c.£1,700. 3D printing a cranium requires extra consideration, not only due to its size (it was too large for several of the printers to print as a single piece) but also due to the large endocranial void. FDM printers will fill this void with a support scaffold (a honeycomb‐like structure) to assist the build process (Fig. 7). An advantage of the SLS technique is that the endocranial void remains true and does not become filled in, indeed SLS does not use any support scaffolds, which can also leave rough surfaces on a print after removal (Fig. 7). Despite the SLA cranium print being split into parts and assembled postprinting, the print does not appear to have been affected in its accuracy which agrees with previous findings stating that 3D scanning and modeling parameters are more important factors than the resolution of a 3D printer 12.

The cheaper FDM printers used in this study (RS and MKB) demonstrated comparable metric error to the costlier SLS and SLA types for the small bones. Although, the use of cheap desktop printers is discouraged by Hodgdon et al. 12, due to users being more likely to incur discrepancies between imaged anatomy and final prints 12. However, given that each of the printers tested here was found to produce accurate prints, it is perhaps more essential to consider the esthetics of the print, the practicality of support structure removal, the production time and the cost when choosing a printer, as these could have a greater effect on the final print quality. SLS was the preferred printing technique as this method: a) produced highly accurate prints; b) exhibited excellent surface quality; and c) does not require support structures during printing.

The durability and quality of a build also need to be considered, for instance, whether the 3D print will alter in size following exposure to UV light, moisture or repeated handling. For example, if storing a 3D printed replica to be used as evidence in court, one needs to be confident that it will not significantly alter in size or appearance during storage, which could be for many years—although of course the replica could be reprinted. Multiple types of materials are available, each with different properties and durabilities. An evaluation of 3D printing material properties for forensic use, bringing together existing engineering and medical expertise with forensic science requirements, is an important next step in evaluating 3D printed osteological replicas. Several of these materials are routinely tested by organizations such as the U.S. Pharmacopeial Convention (USP) Class VI or International Standards Organisation (ISO) 36.

This research evaluated and quantified metric errors throughout the production process and found them to be acceptable (±2.0 mm) under the circumstances tested, especially when other factors that are inherent within osteometric data collection, such as the measurement precision of the instruments and observer error, are considered. Further study with a larger sample size and a focus on one virtual model and one or two printers would be beneficial, including the calculation of the technical error of measurement (TEM) for comparison with the accepted ranges published by Langley (2018) 34.

This preliminary study has laid a foundation for the validation of the 3D printing process and established that SLS 3D prints were the most realistic from the types tested. Additionally, this has provided a useful insight into the capabilities of 3D printers for printing osteological samples, and highlighted that careful consideration is required when selecting scanning and reconstruction parameters. At present, 3D printed human remains should only be presented as demonstrative evidence in court in conjunction with additional existing evidence 5, such as CT images. In order for these new technologies used in the imaging, modeling, and printing of forensic exhibits to be sufficiently robust to stand up to cross‐examination in court, an empirical evidence‐base needs to be formed to underpin the accuracy and reproducibility of the 3D print exhibit.

Further work will explore 3D printer capabilities for printing forensic case specimens exhibiting trauma and fine details, and crucially an experimental investigation into the evidential impact of using 3D techniques for demonstration of evidence. The issues surrounding the validity and reliability of printed replicas and their evidential value must be addressed urgently, to avoid a lack of transparency in evaluative interpretation and the risk of misleading evidence creating unsafe rulings 23, 49.

## Conclusions

This empirical study has shown that the 3D prints created were accurate to the source bone, and provided new data addressing the issue of comparing different printing methods and tracking accuracy throughout the production process. These findings demonstrate that:

- It is possible to produce accurate 3D printed replicas from CT scanned skeletal elements;
- Each printer tested produced replicas with mean differences within ±1.2 mm
- SLS was the most metrically accurate printer type used and produced prints that were the most esthetically true to the original specimen.

Recommendations for 3D printing osteological demonstrative evidence include employing the highest CT scan resolution possible, using a high/hard CT reconstruction filter, applying an appropriate degree of surface smoothing and using a 3D printer that does not require support structures. This research initiates the validation of 3D printed forensic anthropological samples as demonstrative evidence in court.
